# Discrimination in relation to parenthood reported by community psychiatric service users in the UK: a framework analysis

**DOI:** 10.1186/1471-244X-13-120

**Published:** 2013-04-20

**Authors:** Debra Jeffery, Sarah Clement, Elizabeth Corker, Louise M Howard, Joanna Murray, Graham Thornicroft

**Affiliations:** 1 Health Service and Population Research Department, Institute of Psychiatry, Kings College London, De Crespigny Park, London SE5 8AF, UK; 2 Section of Community Mental Health, PO29, Health Service and Population Research Department, Institute of Psychiatry, King's College London, De Crespigny Park, London SE5 8AF, UK

**Keywords:** Discrimination, Stigma, Pregnancy, Parenting, Mental disorders, Mothers, Fathers

## Abstract

**Background:**

Experienced discrimination refers to an individual’s perception that they have been treated unfairly due to an attribute and is an important recent focus within stigma research. A significant proportion of mental health service users report experiencing mental illness-based discrimination in relation to parenthood. Existing studies in this area have not gone beyond prevalence, therefore little is known about the nature of experienced discrimination in relation to parenthood, and how is it constituted. This study aims to generate a typology of community psychiatric service users’ reports of mental illness-based discrimination in relation to becoming or being a parent. A secondary aim is to assess the prevalence of these types of experienced discrimination.

**Methods:**

In a telephone survey 2026 community psychiatric service users in ten UK Mental Health service provider organisations (Trusts) were asked about discrimination experienced in the previous 12 months using the Discrimination and Stigma Scale (DISC). The sample were asked if, due to their mental health problem, they had been treated unfairly in starting a family, or in their role as a parent, and gave examples of this. Prevalence is reported and the examples of experienced discrimination in relation to parenthood were analysed using the framework method of qualitative analysis.

**Results:**

Three hundred and four participants (73% female) reported experienced discrimination, with prevalences of 22.5% and 28.3% for starting a family and for the parenting role respectively. Participants gave 89 examples of discrimination about starting a family and 228 about parenting, and these occurred in social and professional contexts. Ten themes were identified. These related to being seen as an unfit parent; people not being understanding; being stopped from having children; not being allowed to see their children; not getting the support needed; children being affected; children avoiding their parents; children’s difficulties being blamed on the parent’s mental health problem; not being listened to; and being undermined as a parent.

**Conclusions:**

This research highlights the need for: greater support for parents with mental illness, those wishing to have children, and those who lose access or custody; services to better meet the needs of children with a mentally ill parent; training about discrimination for professionals; and parenting issues to be included in anti-stigma programmes.

## Background

Stigma has been conceptualised as comprising ignorance (lack of knowledge), prejudice (negative attitudes), and discrimination (negative behaviour) [1] Discrimination occurs when a person is treated unfairly because of an attribute such as mental illness. Experienced discrimination refers to an individual’s perception that they have been treated unfairly and is an important recent focus within stigma research [2]. It is distinct from the concept of self/internalised stigma as the former relates to an individual’s perceptions about how they have been treated by others (e.g. ‘People treat me like a bad parent’) and the latter to how individuals see themselves (e.g. ‘I see myself as a bad parent’). It differs from prejudice as it is focuses on behavioural, rather than attitudinal, components of stigma.

To our knowledge, four studies have assessed the prevalence of the concept of experienced discrimination due to mental illness across a broad range of life areas, including starting a family and in the role as a parent. Prevalence varied with condition, context and time frame and was 6–40% for discrimination in starting a family and 14–45% for discrimination in the parenting role [2-5]. For parenthood the studies did not go beyond prevalence. Therefore key unanswered questions include: what is the nature of experienced discrimination in relation to parenthood, and how is it constituted?

The majority (60–70%) of women with serious mental illness are mothers [6,7] with fertility rates increasing [8]. The figures are lower for men with 20–30% of men with severe mental illness being fathers [9,10]. Existing studies about the experience of mothering in the context of mental illness [11-13] indicate that stigma is central to the experience for many women. Indeed, a recent systematic review and meta-synthesis of the qualitative literature on pregnancy and motherhood for women with severe mental illness found stigma to be a major theme [14]. In particular, mothers with mental illness often report that they are perceived as bad parents and concern about custody loss is common [12,15-17]. The systematic review [14] made no mention of discrimination or unfair treatment suggesting that this dimension of stigma is rarely addressed in relation to parenthood. Stigma has been identified as an important barrier to care seeking in mothers experiencing mental health problems [18,19]. Stigma also appears in research on the experiences of fathers with mental illness [11,20], although the literature on this is sparse. There is limited literature exploring experiences of stigma and discrimination in women and men considering having children or in pregnancy, although there is some evidence of the desire to be a parent being undermined by societal attitudes for individuals with mental illness [15]. Existing research has also demonstrated that the stigma of parental mental illness can affect children [21]. These studies are informative about the parents’ experiences of the broader concept of stigma. However, none has investigated the specific concept of experienced discrimination (reports of perceived unfair treatment) in relation to parenthood.

It is important to understand the nature of experienced discrimination because parenthood is a key life role, and people with mental health problems should not be discriminated against in this role [22]. The right to family life is enshrined in the UN Convention on the Rights of Persons with Disabilities, which includes reference to people with mental health-related disabilities [23]. In this context, the aim of this study is to generate a typology of community psychiatric service users’ reports of mental illness-based discrimination in relation to becoming or being a parent. Secondary aims are to identify which type of individuals or institutions act in ways experienced as discriminatory; and to assess the prevalence of parenthood-related mental illness-based discrimination

## Methods

### Design

This study is a thematic analysis of qualitative parenthood-related data from a larger telephone interview surveys conducted in 2009 and 2010 [24,25] as part of the evaluation of England’s anti-stigma programme, *Time to Change,* full details of which are given elsewhere [4,26]. The present study examines data from a subset of the survey participants and is therefore a nested study.

### Participants

The survey sample consisted of adults aged 18–65 under the outpatient care of National Health Service community mental health teams. The teams were in ten Mental Health Trusts (five in each survey wave) across England, with the Trusts selected to represent the range of socio-demographic deprivation. There were 1047 participants in 2009 and 979 in 2010. As the surveys at the two time points used independent samples the respondents were combined, and formed a total survey sample of 2026. Survey response rates were 7.0% and 7.6% respectively. The sample as a whole was under representative of men and people from ethnic minority backgrounds. The participants in the present study (n = 304) were a subset of the total sample and were all those who reported having experienced some level of discrimination in relation to parenthood (starting a family and/or being a parent) in the previous 12 months, and gave an example of this. The sample included both mothers and fathers and those who were not parents but had experience of wishing or try to start a family.

### Procedure

A random sample of 4000 people who use mental health services within each of the five Trusts were selected using a software programme in 2009 and again in 2010. Those judged by the mental health team to be at risk of distress from receiving an invitation to participate were excluded, and the remainder were mailed invitations to participate in the study, and were asked to send back a consent form. Each participant was then contacted by telephone to arrange an interview time and formally consent. This two-step process may be one reason why the response rate is low for both years. Interviews were conducted by telephone by one of a panel of telephone interviewers, over two thirds of whom had personal experience of mental illness. Interviewers made a verbatim (or summarised typed) record of reported examples of discrimination. The survey was approved by the Riverside NHS Ethics Committee (07/H0706/72).

### Measure

The main measure in the survey, and the only one of relevance here, is the DISC-12 [27]. The measure has good test-retest reliability and inter-rater reliability. It has also been demonstrated to have convergent validity as it was found to correlate with two other measures of discrimination experience and has divergent validity as it had no significant association with gender as predicted [27]. Participants are asked whether, in the previous 12 months, they have been treated unfairly because of mental health problems in relation to 22 life domains, such as ‘making or keeping friends’; ‘finding a job’; ‘getting help for physical health problems’. Response categories were ‘not at all’, ‘a little’, ‘moderately’ and ‘a lot’ (there is also a ‘not applicable’ option). If the interviewee reports any level of discrimination they are asked to give an example. The data from these examples are the focus of this study. DISC items relevant to the present study are: 1) ‘Have you been treated unfairly in starting a family or having children?’; and 2) ‘Have you been treated unfairly in your role as a parent to your children?’. Respondents were also asked to state their gender, ethnicity and clinical diagnosis.

### Analysis

The prevalence of experienced discrimination in relation to starting a family and in relation to the parenting role was calculated using SPSS v19. A framework analysis [28] was conducted on the typed examples of parenthood-related discrimination. This analysis method is an established and rigorous five-stage method for analysing qualitative data, developed by social research organisation Natcen (http://www.natcen.ac.uk/) and is widely used in social policy and health services research [29]. In the first stage-t*he Familiarisation Process*-DJ and SC read all the examples with the study’s aims in mind and noted the main themes that appeared to recur. In stage two-*Developing a Theoretical Framework*-DJ collated recurring themes into groups of similar themes and organised these into a draft theoretical framework or index which was refined in discussion with SC. Next, in the *Indexing stage,* DJ coded the data, matching it to the themes in the provisional theoretical framework using NVivo 9 software (QSR International) revising or merging themes and creating new categories as necessary. In stage four-*Charting*-data were summarised in thematic charts in an Excel spreadsheet. Each example, or part-example, was included under one or more theme in the thematic chart or under an ‘other’ category to ensure that the dataset was understood as a whole. This process was undertaken independently by DJ and SC who met to agree any disparities. In the final stage-*Synthesising the data;* five team members (DJ, SC, JM, LH and EC) met to map*,* interpret and synthesise the data through reviewing the charted data, comparing themes (distinct concepts) and sub-themes (concepts that were part of a larger theme) with each other, merging, splitting and re-naming these as required. A schematic diagram representing a typology of experienced discrimination in relation to parenthood was produced grouping similar themes together, and based on the best fit to the data, and the final conceptual model agreed. Typical examples were selected to illustrate each theme and subtheme.

## Results

By examining the number of survey participants who reported that the ‘role as a parent’ life domain was applicable to them (1047/2026, 51.6%) we can estimate that approximately half the sample were parents. After excluding those who said the life domain was not applicable to them, 22.5% (196/873) reported being treated unfairly in starting a family due to their mental illness. With regard to being a parent 28.3% (296/1047) reported being treated unfairly in the past year. Twenty seven participants had experienced discrimination in both domains during this time frame. A total of 304 survey participants were eligible for the framework analysis as they reported experiencing some level of mental illness-based discrimination in the previous 12 months regarding starting a family (n = 89), and/or being a parent (n = 215) and gave an example of this. The characteristics of the framework analysis participant’s characteristics are shown in Table 1. The participants gave a total of 317 examples of experienced discrimination, 89 in relation to starting a family and 228 about being a parent.

**Table 1:** Characteristics of participants in the framework analysis sample

**Characteristic**	**Frequency (percentage) (N = 304)**
**Year of participation**	
2009	155 (51.0)
2010	149 (49.0)
**Gender**	
Female	223 (73.4)
Male	80 (26.3)
Transgender	1 (0.3)
**Ethnicity**	
White, British	256 (84.2)
White, other	16 (5.3)
Black, or mixed black and white	3 (1.0)
Asian, or mixed Asian and white	11 (3.6)
Other mixed	4 (1.3)
Did not wish to disclose	14 (4.6)
**Self-reported diagnosis**	
Schizophrenia	33 (10.8)
Schizoaffective disorder	7 (2.3)
Bipolar disorder	80 (26.3)
Depression	83 (27.3)
Anxiety Disorder	12 (3.9)
Personality disorder	25 (8.2)
Other/multiple diagnoses/not stated	64 (21.1)

**Figure 1:**
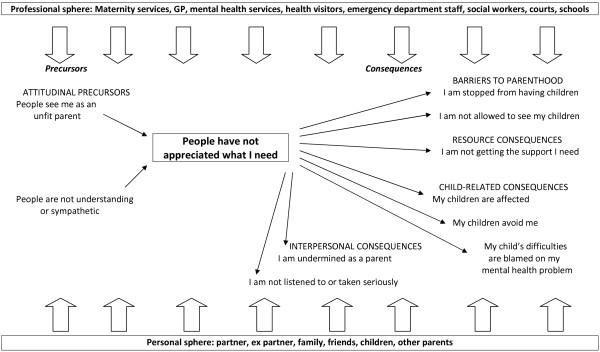
Community psychiatric service users’ reports of discrimination in relation to parenthood: conceptual model and typology.

A final ten-theme conceptual model was constructed from the data, as shown in Figure 1. Two themes were classified as precursors and eight as consequences, and three themes had subthemes. The ten themes were further grouped into five superordinate themes. A core theme of *People have not appreciated what I need* was agreed.

In nearly all themes respondents experienced stigma and discrimination from both the professional and personal spheres of their life. The professional sphere included maternity services, family doctors (GPs), mental health services, health visitors, emergency department staff, social workers, courts and schools. The personal sphere involved partners, ex-partners, friends, family, children and other parents.

The first of two precursor themes-both attitudinal-was *People see me as an unfit parent.* This was the largest theme and incorporated four subthemes. The participants in this theme spoke about how people from both spheres perceived they would be, or were, incapable and inadequate in a parental role. The subthemes in this theme were: *People assume I’m not safe or trustworthy; They come in guns blazing* (reflecting *p*articipants feeling that professionals were being overly protective); *I am judged; and Others make decisions* (reflecting a view that other people made decisions about their children and they were not consulted). The second precursor theme was *People are not understanding or sympathetic.* The themes, subthemes and typical quotes can be seen in Table 2.

**Table 2:** Reported experiences of mental illness-based discrimination in relation to parenthood: illustrative examples

**Themes and subthemes (themes in bold)**	**Illustrative examples**^**a**^
**1) People see me as an unfit parent**	*‘I have been made to feel inadequate as a parent.’*
	*‘There's immediate suspicion from people you're not capable of looking after a child.’*
	*‘The psychiatrist was talking about bringing in social services-that was unfair to assume I can't look after my children.’*
People assume I am not safe or trustworthy	*‘I'm classed as depressed so people won’t let me look after the children. I'm treated like a leper. They assume I'm unsafe or not trustworthy.’*
	‘*Because of my mental illness social services thought I'd burnt my daughter but I hadn't.’*
	*‘When my daughter was a toddler and fell off her space hopper onto a concrete floor, the hospital staff kept asking me 'all these questions', because I was 'a single mother and a mental health parent'. They put my daughter on the ‘at risk’ register and missed the fact that my daughter had a heart problem.’*
They come in guns blazing	*‘People watch me too closely.’*
	*‘Social services are always involved, coming in guns blazing.’*
	*‘I always feel that people are checking up on me and my children because of my diagnosis.’*
	*‘I was kept in hospital for an extra week after having my children because the staff felt they needed to contact social services.’*
I am judged	*‘I feel people were judging me when I was pregnant and having my baby. The midwife for example seemed to jump to conclusions because of my diagnosis.’*
	*‘My son has ADHD, when he's struggling, teachers immediately ask about my mental health and I feel I am being judged’.*
	*‘I feel that other parents judge me.’*
Others make decisions	*‘My mum recently took my son to Sweden (which was good) but she didn't ask me, and I didn't know about it, and I'm his mother after all.’*
	*‘They [social services] take your power to make decisions because they think you aren't thinking straight.’*
**2) People are not understanding or sympathetic**	*‘Mental health staff said I should pull myself together because I have a family’.*
	*‘My daughter’s college made me feel like an irresponsible as they did not understand that I could not speak to them at that time.’*
	*‘The school has not been sympathetic when my son is late to school or not handed things in (*e.g. *money).’*
	*‘She was not understanding of my situation and was making inappropriate demands of me.’*
**3) I am stopped from having children**	*‘We were basically told don't even go there by the hospital and a psychiatrist and that we wouldn't be considered for adoption.’*
	‘*A doctor advised me not to have children, it was the worst thing I could do.’*
	*‘My family said I should never marry or have children.’*
I am not helped to come off or change my medication to have children	*‘Nobody wanted to help me to get off tablets to enable a pregnancy.’*
	*‘I've been told I have to stay on the medication I've been given and also told that the medication causes birth defects’.*
	*‘I can’t conceive because of medication.’*
	*‘I have a fiancé and we would like to get married and have children but again the medication interferes and I don't want to have a disabled baby.’*
I was told I wouldn’t be able to adopt	*‘I have children and I'm trying to adopt another but mental health could be an issue apparently, even though I've got children of my own and brought them up.’*
	*‘We considered adoption but I was told that with my mental health issues, it would be unlikely.’*
They didn’t want to give me IVF	*‘My fiancé could not have children so we looked into IVF. My doctor was funny about supporting me and discouraged me from having children.’*
	*‘I've been told I'll find it hard to conceive because of my medical problems and mental health problems and that adoption/IVF treatment is out of the question.’*
People discourage me from having children because they think I will relapse	*‘I feel my psychiatrist is frightened I will become ill again.’*
	*‘I can’t conceive because of my medication and I’m not able to have children because my illness would get worse.’*
If I have a baby it would be taken away	*‘I was told if I had a baby I would be taken.’*
	*‘If I want a family I am likely not to be able to keep any children and am worried about it’*
**4) I am not allowed to see my children**	
My children have been taken away	*‘My children have been taken away and I can't put my point across 'cause no one believes me.’*
	*‘Police took my kids away from me when they were young.’*
	*‘I had two babies, one was taken away.’*
	*‘After my divorce my children were taken by my ex-husband one by one.’*
I am not allowed to be alone with my child	*‘I am not able to see my children unsupervised.’*
	*‘I’m not allowed to have my children while my husbands at work.’*
My ex-partner makes it difficult to see my child	*‘I have access to my son but the mother has taken him to live in Glasgow-she knows it’s difficult for me to go on a train so I can’t see him very often.’*
	*‘I gained access to my 10 year old son 3 or 4 weeks ago. My wife took him away from me 2 years ago because of my mental health.’*
	*‘I have 4 children-they are very young. My ex wife doesn't want me to see them. I saw a solicitor but they said I had no chance. It’s unfair on me and the children.’*
I am not allowed to be a grandparent	*‘I am never allowed to babysit my grandchildren and I am very much withheld from seeing them.’*
	*‘I’m not allowed to have my grandson on his own.’*
I’ve had problems getting my children back	*‘I put my children in voluntary care and now I’m having difficulty getting them back.’*
	*‘I had to fight to get one back after hospital, it took about three months’*
People used my mental health against me	*‘Social services would take my baby away if I had another. They took my son at 4 months old, even though I was a good mother. They used my history of mental health against me.’*
	*‘Social services have not given me a chance and are using my mental health problems against me.’*
**5) I am not getting the support I need**	*‘I have had no social services support in maintaining my family.’*
	*‘I have had to accept that I will never have children, but there has been no support offered in dealing with this very difficult thing.’*
	*‘I feel I'd have benefitted from a little more support. There's no back up if I can't take care of my children.’*
**6) My children are affected**	*‘When I was last ill I upset my son’s friend’s mother and he hasn't been invited to events because of that.’*
	*‘My daughter was bullied a school because of my diagnosis.*’
**7) My children avoid me**	*‘My daughter tended to avoid me and kept saying 'be happy'.’*
	*‘They don't ring me or keep in touch, they just ignore me’*
	*‘They don't involve me in any of their decisions and never ask me for advice/opinions any more.’*
**8) My children’s difficulties are blamed on my mental health**	*‘We're currently having problems with our 15 year old daughter and the social services say it's all because of my mental health-I feel they're saying it's my fault and that's unfair.’*
	*‘If my son doesn't gain weight the health visitor thinks it’s due to my mental health.’*
**9) I am not listened to or taken seriously**	*‘I don't feel the school listens to us as parents. I'm asking for a dyslexia assessment for my child but the school think it's something to do with my mental health.’*
	*‘When I discuss the problems about my children, the doctors don't listen to me as a parent, because of my mental health.’*
	*‘When I try and reason with my ex husband he brings up my mental health.’*
**10) I am undermined as a parent**	*‘My diagnoses seem to get in the way of my rights as a parent.’*
	*‘My ex-wife belittles me in front of children.’*
	*‘My husband threatens to tell the children I am mad*.’

^a)^ Examples recorded in the third person have been amended to the first person for consistency of presentation.

The two largest consequence themes were both about barriers to parenthood: *I am stopped from having children* and *I am not allowed to see my children*. Respondents spoke about both professionals and family being disapproving or discouraging about them starting a family. Subthemes related to not being helped to come off or change medication to enable a pregnancy, lack of access to IVF and adoption, discouragement due to relapse concerns, and not being able to have a child due to fears or knowledge that the baby would be taken away from them.

The theme of *Not being allowed to see my children* was reported by both mothers and fathers, and was the largest theme for male respondents although men were represented in all other themes too apart from one (*My children are affected*). The subthemes in the category *I am not allowed to see my children* related to children being taken into care by social services or no longer seeing them because of loss of custody to ex-partners or to family members. Participants also spoke about feeling unfairly treated by having restricted access to their child due to not being allowed to be alone with them or by ex-partners making access difficult. Some described how they were stopped from seeing their grandchildren or undertaking usual grandparent roles with them such as caring for them alone. Participants also described having problems and delays in getting their children back after periods in care during illness episodes. The final subtheme relating to seeing ones children was about people using participants’ mental health against them to gain access or custody of their children.

The remaining six themes did not have subthemes. The largest theme without subthemes was *I am not getting the support I need* and focused mainly on a lack of support from social services and in health care as well as there being little support available beyond social services. Three themes were child-related. One related to participants’ children being treated negatively because of their parent’s mental illness. Another was about the participants’ own children treating them unfairly, specifically a tendency for them to avoid the parent. A further issue relating to the children was the children’s health, behavioural and educational difficulties being blamed on the parent’s mental illness. The two final themes were interpersonal. In one participants believed they were not listened to or taken seriously as parents, particularly by the school, by doctors, partners and children. The other theme was about the parents feeling that they were undermined in their role as a parent either by the children themselves, ex-partners or by other relatives.

## Discussion

This study is the first to investigate the nature of service user-reported experiences of mental-illness based discrimination specifically in relation to parenting. We have produced a detailed typology of reported unfair treatment regarding becoming or being a parent through a rigorous iterative process. Although participants were asked about ‘being treated unfairly’ as we were seeking to understand experienced discrimination which is conceptualised as a behavioural construct [1], two of the themes, both precursors, were attitudinal, reflecting the prejudice component of stigma: ‘People see me as an unfit parent’ and ‘People are not understanding or sympathetic’ unfair treatment’. The remaining themes related to consequences, including precluding parenting, low levels of support, and interpersonal and child-related consequences. Further, we have identified the many professional and social groups seen as behaving in discriminatory ways.

The findings of this study support the literature on the topic. Parents with mental illness often report that they are perceived as bad parents [11,12,15-17]. This study provides further evidence on the prevalence of parenthood-related discrimination due to mental ill health and extends existing research [2-5] by going beyond prevalence to examining the nature of this phenomenon. Our study addressed the under-researched area of stigma and discrimination in relation to starting a family. To our knowledge no previous studies have reported mental illness-based unfair treatment regarding IVF and adoption.

This study included men as well as women and thus contributes to our understanding the experience of fathering in the context of mental illness. Like other research [30], we found that access and custody were major issues for fathers with mental illness, but also discovered eight other areas where men reported parenthood-related experienced discrimination. Our research supports Ackerson’s [21] in finding reports of the stigma of parental mental illness extending to affect children. Also, parents in this study reported being avoided by their own children, a scenario which might have been prevented through better support for the needs of children in this situation [31]. For some parents, experienced discrimination extended into their roles as grandparents, a phenomenon not previously noted in the literature.

### Strengths and limitations

This study is novel as it is the first to describe the nature of ‘experienced discrimination’ in relation to parenthood. It includes a large geographically diverse sample. Respondents were asked about many life areas therefore the study sample is not biased towards those who feel they have been treated unfairly regarding parenthood. A further strength is that the study includes responses from fathers and participants who are grandparents. The analysis was rigorous and involved multiple researchers to enhance the trustworthiness of the findings.

There were some limitations in the original survey [4,24,25] that therefore also affect this study. Due to the nature of the survey there was a lack of probing and follow-on questions for the examples that participants provided of their experiences. There was a low response rate to the survey and the possibility of non-representativeness as the people who were surveyed had put themselves forward for the research, and men and those from ethnic minorities were under-represented. Furthermore, the time frame for the survey questions was restricted to experienced stigma and discrimination in the previous 12 months, therefore important past experiences may have been missed, particularly those relating to past pregnancies or attempts to start a family. Experiences are inherently subjective and may not fully reflect actual events or practice. Participants were only asked about unfair treatment and we did not investigate examples of experienced non-discrimination and positive discrimination so the findings may not give a rounded picture of experiences. Because of these limitations the findings must be viewed as somewhat tentative.

## Conclusions

### Implications for research

Future research in this area can include face-to-face interviews, more probing and longer qualitative interviews, and focus group studies to explore issues in greater depth and build on the findings presented here. We also advocate large quantitative studies of representative samples focusing on experienced discrimination in relation to parenthood to establish the prevalence of the experiences described in each theme and subtheme. Such research could also investigate which groups are most likely to experience discrimination in relation to parenthood. The themes and subthemes may be used as a basis for constructing a measure to assess people’s experiences of discrimination regarding becoming or being parents. There is a particular need for more research on the needs of women who have lost custody of their children or are facing this situation, and to establish the best ways to support them to prevent this happening. Research is also needed to establish the best ways to support fathers with mental illness to maintain their relationships with their children.

### Implications for policy and practice

This research highlights a vital need for changes in policy and practice, a major one being for more support for parents with mental illness [32-34]. Our findings also suggest that parents have sometimes been given unhelpful and possibly inaccurate advice, for example that they cannot conceive as they have to stay on the medication and that it may cause congenital abnormalities. If true it indicates they have not had a detailed pre-conception counselling involving a full discussion of risks and benefits of the various options available regarding medication [35] . The large theme on access on and custody indicates a need for services to better support parents around these matters to ensure inappropriate separations and their ramifications are avoided [36,37]. Services could help to ensure equity and parity for prospective and current parents with mental health problems and other parents or parents with physical disabilities through awareness of human rights legislation regarding family life [23], and through conceptualising support services as a form of reasonable adjustments or accommodations [22]. Incorporating information about avoiding discrimination into professional training courses may be warranted. Although there is an increasing emphasis on the needs of children with mental health problems [38,39] and a number of innovative approaches to supporting them have been developed [40,41], it is clear from our findings that there is a need for more and better support for these children. Public anti-stigma campaigns can specifically address societal attitudes to parents with mental illness, which may ameliorate some of the discrimination experienced in the social as well as professional spheres. Key implications for clinical psychiatrists centre on (i) providing, or referring for, supportive, objective pre-conception/pregnancy counselling patients considering becoming parents or expecting a baby; (ii) ensuring patients who are parents, and their children, receive for the best available supportive services to meet their various and changing needs; (iii) being aware of the multitude of negative assumptions and behaviours that female and male patients face in relation to parenthood, challenging these as appropriate, and talking with patients about their experiences of discrimination in relation to parenthood to acknowledge the experiences and identify ways to help.

## Competing interests

The authors declare that they have no competing interests.

## Authors’ contributions

SC designed and supervised the study and co-analysed the data. DJ led on the literature review and analysis. EC and GT led the study that provided the data for the present study, and EC extracted these data. LH, JM and EC met with DJ and SC to review and discuss the data, identifying themes and developing the conceptual model. GT contributed to data interpretation and to the final conceptual model. DJ and SC drafted sections of the manuscript and SC finalised the draft. All authors critically revised the draft manuscript, and read and approved the final manuscript.

## Pre-publication history

The pre-publication history for this paper can be accessed here:

http://www.biomedcentral.com/1471-244X/13/120/prepub
